# Impact of Exercise Regimens on Childhood Obesity Through Gut Microbiota Regulation: A Systematic Review

**DOI:** 10.1155/ije/9999960

**Published:** 2026-07-13

**Authors:** Si Zhang, Fei Han, Bo Wang, Zilin Chen, Jing Lyu, Fei Fan

**Affiliations:** ^1^ Suzhou TCM Hospital Affiliated to Nanjing University of Chinese Medicine, Suzhou, China, njucm.edu.cn; ^2^ Guang’anmen Hospital, China Academy of Chinese Medical Sciences, Beijing, China, cacms.ac.cn

**Keywords:** childhood obesity, exercise, gut microbiota, mechanism

## Abstract

Childhood obesity represents a global concern, adversely affecting the physical and mental health of children. This condition results from a complex interplay of genetic and environmental factors, among which the gut microbiota plays a significant role. Exercise, a crucial lifestyle factor, impacts obesity, with the gut microbiota potentially serving as a mediating link between the two. Numerous clinical studies have demonstrated that physical activity can modify microbial imbalances and the metabolic networks of the gut microbiota, thereby effectively addressing childhood obesity. However, the precise molecular mechanisms involved remain inadequately understood. This review aims to investigate the impact of exercise on the gut microbiota and the microbial metabolites it produces. In addition, we provide a summary of several clinical trials from the past two decades that have examined how exercise influences the gut microbiota and affects the progression of childhood obesity. This study offers both theoretical and practical insights for comprehensive interventions targeting childhood obesity.

## 1. Introduction

According to the WHO, overweight and obesity are characterized by an abnormal or excessive accumulation of fat that poses a health risk [1, 2]. In 2019, the World Obesity Federation projected that by 2030, there would be 254 million children and adolescents aged 5–19 years affected by obesity [3]. Obesity in childhood often continues into adulthood, leading to substantial lifetime expenses and elevating the likelihood of developing other conditions such as coronary heart disease, stroke, and diabetes [4, 5]. The intestinal microbiota, which is the collective assembly of microorganisms residing in the gastrointestinal tract, is essential for human metabolism. The gut microbiota and their metabolites have been identified as key contributors to the metainflammation seen in obesity. Numerous studies have demonstrated that imbalances in the gut microbiota impact metabolic and cognitive functions and are closely linked to obesity [6]. In cases of childhood obesity, there is a noted decrease in Bacteroidetes and an increase in Firmicutes within the gut microbiota, which enhances the ability to extract energy from carbohydrates [7, 8]. In addition, the diversity of gut microbiota in children with obesity differs from that of their normal‐weight counterparts [9]. Microorganisms within the gastrointestinal tract adjust their metabolic processes, shift in dominance and population, and even undergo genetic changes to optimally exploit specific microenvironments [10, 11]. Lifestyle elements, including diet, physical activity, sleep, and stress levels, significantly influence the gut microbiota [12, 13]. In a 12‐week randomized controlled trial [14], rope skipping notably decreased the relative abundances of *Lactobacillus* and Muribaculaceae, while a high‐fiber diet led to a reduction in *Lactobacillus* and *Eubacterium* populations. Engaging in physical exercise at a suitable frequency and intensity boosts the production of short‐chain fatty acids (SCFAs), a microbial metabolite that activates AMP‐dependent kinase (AMPK) and improves energy metabolism [15]. Alterations in the gut microbiome can affect children’s sleep patterns through immunomodulatory and metabolic pathways, potentially resulting in additional health issues such as obesity [16]. Stress can influence the gut microbiome, potentially affecting chronic inflammation and metabolic processes associated with obesity [12, 17].

This systematic review contributes to the existing literature by focusing on the pediatric population and synthesizing evidence on exercise‐inclusive lifestyle interventions—which often include concurrent dietary modifications—and their effects on the gut microbiota, an area with limited dedicated synthesis.

## 2. Methods

### 2.1. Study Design

This systematic review was performed following the Preferred Reporting Items for Systematic Reviews and Meta‐Analysis 2020 (PRISMA 2020) statement [18]. The analysis methods and inclusion criteria have been documented in a protocol published in the Prospective International Register of Systematic Reviews (PROSPERO) under the registration number CRD420261404161, available from https://www.crd.york.ac.uk/PROSPERO/view/CRD420261404161. The completed PRISMA 2020 checklist is provided in Supporting Information 2.

### 2.2. Search Strategy

We performed a search of the PubMed electronic database from January 1993 to August 2025, restricted to studies published in English due to resource constraints. Our search strategy included a wide range of terms related to childhood obesity and exercise‐related lifestyle, emphasizing their interactions and possible mechanisms involving the gut microbiota. Boolean operators AND and OR were used to combine search terms. The complete search strategy is presented in Table 1.

**TABLE 1:** Search strategy for MEDLINE (PubMed) database.

Query	Records retrieved
(“obesity”[Mesh] OR “overweight”[tiab] OR “overnutrition”[tiab]) AND (“child”[Mesh] OR “adolescent”[Mesh] OR “children”[tiab] OR “pediatric”[tiab] OR “paediatric”[tiab] OR “youth”[tiab] OR “teen”[tiab] OR “teenager”[tiab]) AND (“gastrointestinal microbiome”[Mesh] OR “microbiota”[All Fields] OR “gastrointestinal”[All Fields] OR “microflora”[All Fields] OR “flora”[All Fields] OR “gut”[All Fields] OR “intestinal”[All Fields]) AND (“exercise”[Mesh] OR “physical”[tiab] OR “sedentary”[tiab] OR “activity”[tiab] OR “training”[tiab] OR “sport”[tiab])	254

### 2.3. Eligibility Criteria

Articles that meet the following criteria of the “Population, Intervention, Control Group, Outcome, and Study Design” (PICOS) framework can be included (Table 2).

**TABLE 2:** Eligibility criteria using the PICOS framework.

Eligibility criteria	Description/characteristics
Population	Individuals with childhood obesity of any age, sex, or ethnicity
Interventions	Must include exercise regimens
Comparators	Studies without a comparator will still be eligible for inclusion
Outcomes	BMI and possible mechanisms involving the gut microbiota
Study design	Case reports, clinical study, clinical trial, observational study, randomized controlled trial

The following types of publications were excluded:

1. Duplicate publications
2. Retracted articles
3. Non‐English–language reviews.

### 2.4. Study Selection

**FIGURE 1:**
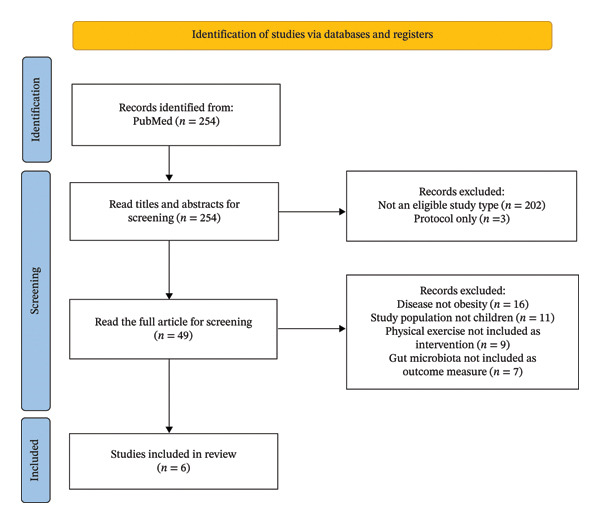
Publication screening process.

All reviewers (Fei Fan, Bo Wang, and Si Zhang) received training on the inclusion criteria prior to the formal review process. Their consistency was assessed using Cohen’s kappa statistic [19] based on 15 randomly selected published articles, with a predefined consistency threshold of *κ* > 0.80. Calibration tests were repeated until this threshold was achieved. All records identified through the search were imported into EndNote X9 (Clarivate Analytics, Philadelphia, PA, USA). Two reviewers (Fei Fan and Si Zhang) independently screened the titles, abstracts, and keywords of the remaining records against the inclusion criteria. Records deemed potentially eligible were retrieved for full‐text assessment. The same two reviewers independently evaluated the full‐text articles for final inclusion, documenting reasons for exclusion at each stage. Any disagreements during the full‐text review were resolved through discussion or by consulting a third reviewer (Bo Wang). The search and screening process was summarized using a PRISMA flow diagram (Figure 1).

### 2.5. Data Extraction

We developed a standardized data extraction form to extract essential data from the included articles. The data extraction was independently completed by two reviewers (Fei Fan and Si Zhang), and cross‐checking was conducted by a third reviewer (Fei Han). The extracted variables included the article title, first author, publication year, country, methodological quality, study design, age range, sample size, intervention, exercise method, and main outcomes. In case of any disagreement during the data extraction process, it was resolved through discussion or by the third reviewer (Fei Han) making the final decision.

### 2.6. Quality Assessment

Two reviewers (Fei Fan and Si Zhang) independently assessed the methodological quality of the included studies. The Cochrane Risk of Bias 2.0 (RoB 2) tool was used to evaluate RCTs [20]. For nonrandomized studies, the RoB in Nonrandomized Studies of Interventions (ROBINS‐I) tool (Version 2) was applied [21]. Any disagreements during the quality assessment were resolved through discussion or by consulting a third reviewer (Bo Wang).

### 2.7. Data Synthesis

The main components of the research results include the following: BMI, possible mechanisms involving the gut microbiota, and the influence of physical exercise on these factors. In instances where the research includes both narrative and quantitative data, quantitative data results should be prioritized for summary. Due to significant heterogeneity in the types of physical exercise interventions, outcome indicators, and participant characteristics across different studies, we did not conduct a meta‐analysis. Detailed characteristics, methodological quality assessments, and main outcomes of all included studies are presented in Supporting Information 1.

## 3. Results

Both aerobic exercise and combined aerobic exercise and strength training significantly decreased BMI Z‐scores [22]. Most studies on exercise interventions in childhood obesity are often combined with dietary interventions (Table 3), making it difficult to assess the effects of exercise alone on the gut microbiota associated with obesity. A randomized controlled trial by Quiroga et al. showed that after physical exercise, the abundance of specific gut microbiota in children with obesity changed toward a profile similar to that of healthy children [23]. In addition, the secretion of obesity‐related inflammatory markers that decreased after the intervention, such as NLR family pyrin domain containing 3 (NLRP3), osteopontin (OPN), and Caspase‐1 (CASP‐1), may be associated with changes in the gut microbiota [23]. Wang et al. introduced the REVERIE system, known in Chinese as “Lingjing,” a virtual reality platform based on reinforcement learning [25]. This system combines the expertise of sports professionals with an AI‐driven personalized program to deliver comprehensive and empathetic guidance on exercise skills for overweight adolescents. Research [25] indicated an increase in alpha diversity following REVERIE interventions. Within the REVERIE exercise group, 23 types of microorganisms showed significant changes, including an increase in the abundance of the Firmicutes phylum and a decrease in the abundance of *Escherichia coli* and microorganisms from the *Klebsiella* genus. *E. coli* and *Parasutterella excrementihominis* were closely associated with changes in neural activity in regions linked to cognition and emotion after REVERIE sports.

**TABLE 3:** Research on the connections between physical activity, gut microbiome, and obesity in children.

Authors/year	Country	Study design	Diagnosis (sample size)/age	Intervention	Main outcomes	Conclusion
Quiroga et al. [23] 2020	Spain	Randomized controlled trial	Obesity (*n* = 39), 7–12 years old	A 12‐week strength and endurance combined training program	*Blautia*↑, *Dialister*↑, *Roseburia*↑	The training program appeared to boost certain genera, including *Blautia*, *Dialister*, and *Roseburia*, which were found to be diminished in patients with obesity, aiming to achieve a microbiota profile akin to that of healthy control children
Nagata et al. [24] 2017	Japan	Clinical trial	Obesity (*n* = 12), average age = 10 years 9 months old	An exercise regimen lasting six months and diet therapy	*Bifidobacterium*↑, acetic acid concentration↑	Although diet and exercise therapy led to improvements in obesity, the benefits were short‐lived due to a subsequent rebound
Wang et al. [25] 2025	China	Randomized controlled trial	Obesity (*n* = 89), average age = 10 years 9 months old	An 8‐week physical intervention using a system named REVERIE	*Streptococcus thermophilus*↑*, Escherichia coli*↓*, Klebsiella genus*↓	13 species showed an increase in abundance, mainly from the Firmicutes phylum. Conversely, 10 species decreased
Cho [26] 2021	Korea	Clinical trial	Obesity (*n* = 36), 7–18 years old	A 2‐month weight reduction intervention of diet and exercise	Bacteroidia class↓, Bacteroidales order↓, Bacteroidaceae family↓, *Bacteroides* genus↓, Firmicutes↑	Changes in lifestyle can influence the composition, diversity, and predicted functional profiles of the gut microbiota in children with obesity
Huang et al. [27] 2020	China	Clinical trial	Obesity (*n* = 24), 9–16 years old	A 6‐week program of combined endurance and strength training exercise and dietary restriction	*Streptococcus*↓, *Veillonella*↓, Christensenellaceae↑	Interventions involving exercise and diet have influenced the gut microbiota composition in children with obesity
Morán‐Ramos et al. [28] 2022	Mexico	Clinical trial	Obesity (*n* = 6), 11–14 years old	A 6‐week dietary and intense physical activity measures	No alterations in BMI, body fat, or the composition and diversity of gut microbiota	The presence of *Odoribacter* is linked to measures of body fat and metabolic health

↑, increase; ↓, decrease.

As discussed above, the baseline gut microbiota composition may serve as a predictive factor for obesity, and individualized profiles of the gut microbiota may partly result in varied responses to the same intervention. In a controlled study conducted by Morán‐Ramos et al., participants underwent a 6‐week dietary and training plan, which resulted in no significant changes in BMI, body fat, or microbial composition and diversity [28]. However, a reduction in waist circumference was observed, and those with a significant reduction in waist circumference showed an increased abundance of *Odoribacter* [28]. This suggests that the dysbiosis of the gut microbiota in overweight individuals may be more challenging to adjust for, although this outcome has not been confirmed in children. When exercise is used as an intervention for obesity, the form, intensity, and duration should be personalized. Nagata et al. [24] used a sequential design: 6 months of diet and exercise alone, followed by 6 months with added *Lactobacillus casei* Shirota (LcS). Diet and exercise alone failed to reduce weight or increase *Bifidobacterium*. After LcS supplementation, weight significantly decreased (−2.9 ± 4.6%), while fecal *Bifidobacterium* and acetic acid increased. This suggests that exercise and diet alone may be insufficient to restore gut microbiota dysbiosis in children with obesity, and that probiotics may be needed for metabolic improvement. Cho et al. [26] stratified participants into fat loss and fat gain groups after a 2‐month lifestyle program. In the fat loss group, Bacteroidetes decreased while Firmicutes increased—opposite to the traditional obesity‐associated profile. Functional prediction (PICRUSt2) revealed that metabolic pathways linked to weight loss and insulin signaling were enriched. These findings show that lifestyle modifications alter gut microbiota in a response‐dependent manner. Huang et al. [27] used a 6‐week intensive program (5 h/day, 6 days/week) combining exercise with dietary restriction. Body weight (−10.5%), BMI, and body fat (−23.4%) significantly decreased. The Firmicutes/Bacteroidetes ratio decreased (from 1.11 to 0.737, *p* = 0.011), and alpha diversity increased. Christensenellaceae (linked to lean phenotype) was enriched. Changes in *Cronobacter*, UCG‐003, and *Helicobacter* correlated with improved central hemodynamics (SEVR + 53%, arterial stiffness, and heart rate).

Of note, although an increased Firmicutes/Bacteroidetes ratio has traditionally been associated with obesity, the findings from Cho et al. [26] and Wang et al. [25] appear to challenge this simplistic view. In both studies, beneficial outcomes (fat loss in Cho et al.; REVERIE exercise intervention in Wang et al.) were associated with increased abundance of certain Firmicutes taxa.

## 4. Discussion

Regular exercise has been associated with altered gut microbial diversity and community composition, which is partly explained by changes in microbial ecology. Physical exercise promotes gastrointestinal motility and the release of neuroendocrine hormones, reduces intestinal blood flow, affects the integrity of the intestinal mucus layer, and regulates the immune system [29]. The gut microbiota network tends to be more robust and connective in active individuals; in contrast, sedentary individuals exhibit reduced diversity and less dense microbial network structures [30]. In insulin‐resistant subjects, exercise training three times a week over two weeks increased the abundance of Bacteroidetes, resulting in a decreased ratio of Firmicutes/Bacteroidetes (which is thought to be associated with obesity) [31]. In another study, after six months of endurance, resistance, and flexibility training, patients with diabetes showed decreased intestinal mycetes, the overgrowth of which correlated with low‐grade inflammation [32]. Exercise can modify metabolites derived from the microbiota and host. Stool butyrate levels increased with exercise in physiologically aging participants [33], and resistance and endurance exercise led to a decrease in serum and fecal bile acids, which interact with the gut microbiota [34].

Different intensities, modalities, and durations of exercise can have distinct effects on the gut microbiota. Compared to habitual living, structured exercise increases alpha diversity and upregulates the metabolic potential of the gut microbiota [35]. Prolonged regular exercise can reshape the intestinal microenvironment of the host; however, acute intense exercise may disrupt gut mucosal barrier function, leading to the formation of a “leaky gut” and triggering inflammatory responses [36]. The modulatory effect of exercise on the gut microbiota may be affected by diet [37]. Dietary intake should be standardized when studying the relationship between exercise and the microbiota.

## 5. Omics Methodologies for Investigating the Gut Microbiota in Pediatric Obesity

In cases of childhood obesity, the gut microbiota often exhibits an ecological imbalance, characterized by diminished diversity, alterations in microbial community structure, and functional disorders. Initial studies predominantly utilized 16S rRNA gene sequencing, which is capable of revealing compositional changes in the microbial community at the phylum and genus levels. However, 16S sequencing does not provide direct insights into the specific functions, metabolic pathways, and active states of the microbial community. To overcome this limitation, omics technologies such as metagenomic, metabolomic, lipidomics and proteomics analysis have been used.

### 5.1. Metagenomic Analysis

In the study conducted by Quiroga et al. [23], metagenomic analysis was utilized to elucidate the bacterial composition associated with obesity at the phylum, class, and genus levels [38]. Concurrently, principal coordinate analysis, based on the Morisita–Horn index, was employed to examine the impact of obesity and exercise on the distribution of microbial communities at the phylum level. Furthermore, phylogenetic analysis of these sequences identified the presence of 549 known bacterial species in fecal samples from both healthy control children and children with obesity. The metagenomic analysis conducted by Wang et al. [25] demonstrated that following the exercise intervention, there was an upward trend in *α*‐diversity. In the REVERIE exercise group, the abundance of 23 microorganisms, representing 12.3% of the common species, exhibited significant alterations. Notably, 13 species with increased abundance were predominantly from the Firmicutes phylum, whereas the microbial species that decreased in abundance included *Escherichia coli* and members of the *Klebsiella* genus, among others.

### 5.2. Metabolomic Analysis

Quiroga et al. [23] conducted a metabolomics analysis utilizing the MetaboAnalyst 4.0 platform [39] to evaluate the metabolic distinctions in the gut microbiota between patients with obesity and healthy individuals. A comprehensive analysis was performed using the PLS‐DA method to detect fecal metabolites, including bile acids, SCFAs, free fatty acids, amino acids, carbohydrates, nucleotides, and organic acids. The findings indicated that exercise modified the metabolic profile of patients with obesity, evidenced by a reduction in branched‐chain amino acids such as isoleucine and leucine, a moderate decrease in formic acid and alanine, and a decrease in xylose, glucose, and galactose. Wang et al. [25] conducted a metabolomics analysis and identified alterations in 42 metabolites following the REVERIE sports intervention. Notably, significant changes were observed in small molecule metabolites associated with lipid metabolism, including chenodeoxycholic acid, acyl‐carnitines, 2‐hydroxybutanoic acid, and 3‐hydroxybutanoic acid.

### 5.3. Lipidomics Analysis

Wang et al. [25] conducted a lipidomics analysis and found that, in comparison to traditional exercise, the REVERIE sports intervention resulted in a significant reduction of various lipids, including triacylglycerols, diacylglycerols, sphingomyelins, and ceramides. In addition, several sphingolipid species, such as ceramide 1‐phosphate (Cer1P), dihydroceramide (DhCer), and glycosphingolipids represented by monosialodihexosylganglioside (GM3), were notably decreased following the REVERIE sports intervention.

### 5.4. Proteomics Analysis

The study conducted by Wang et al. [25] demonstrated that regular physical exercise resulted in significant alterations in 47 proteins, representing 5.6% of the total proteins analyzed. In contrast, the REVERIE exercise led to significant changes in 12 proteins, accounting for 1.4% of the total analyzed proteins. Consistent with previous research [40], the proteins that were upregulated following physical exercise were predominantly involved in processes such as hydrogen peroxide decomposition metabolism (CAT and PRDX2) and oxygen transportation (HBA1, HBB, and HBD). Conversely, proteins associated with cell adhesion (ICAM1 and ITGA2B) were specifically downregulated postexercise.

## 6. Conclusion

**FIGURE 2:**
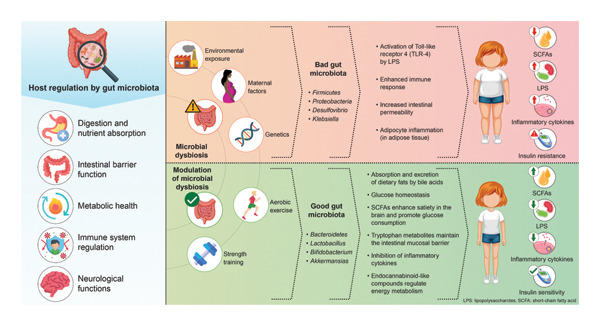
The interplay between exercise, gut microbiota, and childhood obesity. Physical exercise modulates gut microbiota composition, promoting a shift from a dysbiotic profile toward a more beneficial microbial community. These changes influence multiple host physiological pathways, including digestion and nutrient absorption, intestinal barrier function, metabolic health, immune system regulation, and neurological functions.

Childhood obesity is associated with the onset of various lifestyle‐related diseases in adulthood. The gut microbiota, a significant environmental factor, plays a crucial role in energy production and the regulation of inflammation, thereby contributing to the pathophysiology of obesity. A pivotal element in the diet–microbiota–host interaction is the production of a unique array of bioactive microbial metabolites [41]. These metabolites function as essential signaling molecules, modulating host physiology by influencing inflammatory pathways, maintaining intestinal barrier integrity, and facilitating gut–brain communication. Ultimately, they direct the overall metabolic trajectory toward either health or obesity. Key microbial metabolites involved in this process include SCFAs, bile acids, lipopolysaccharide, tryptophan metabolites, and TMAO. Through the utilization of omics technologies, including metagenomics, metabolomics, lipidomics, and proteomics analysis, researchers have acquired a more comprehensive understanding of the specific functions, metabolic pathways, and active states of microbial communities (Figure 2).

The majority of research on the relationship between obesity and gut microbiota has been conducted in adults, with relatively limited data available on children. Furthermore, studies focusing exclusively on exercise as an intervention method are scarce. Exercise methods incorporating virtual reality are more appealing to children, can enhance the motivation of adolescents with obesity to engage in physical activity, and hold considerable potential as a treatment strategy for adolescent obesity. However, the high development costs and challenges in widespread adoption present significant obstacles to their popularization.

## Author Contributions

Fei Fan and Fei Han designed the research study. Bo Wang, Zilin Chen, and Jing Lyu performed the research. Si Zhang and Fei Fan wrote the manuscript. All authors contributed to editorial changes in the manuscript.

## Funding

This study was supported by the Beijing Traditional Chinese Medicine Science and Technology Development Fund Project (Grant no. BJZYZD‐2023‐09) and the Fundamental Research Funds for the Central Public Welfare Research Institutes (Grant nos. ZZ17‐XRZ‐043, 44002, and ZZ15‐XY‐PT‐03).

## Disclosure

All authors read and approved the final manuscript.

## Ethics Statement

The authors have nothing to report.

## Consent

The authors have nothing to report.

## Conflicts of Interest

The authors declare no conflicts of interest.

## Supporting Information

Additional supporting information can be found online in the Supporting Information section.

## Supporting information


**Supporting Information 1** Summary of included studies. A structured table detailing the characteristics, methodological quality assessment, and main outcomes for all six studies included in the review. Available in the supporting file Supporting_Information_1‐Formatted_Data.xlsx.

**Supporting Information 2** PRISMA 2020 checklist. A completed checklist documenting the adherence of this systematic review to the PRISMA 2020 guidelines, with item‐by‐item reporting locations specified. Available in the supporting file Supporting_Information_2‐PRISMA_2020_checklist.docx.

## Data Availability

The datasets used and analyzed during the current study are available from the corresponding author on reasonable request.
